# Development of a Quantitative Diagnostic Criterion for Gastric Linitis Plastica: Findings From a Large Single-Institutional Study

**DOI:** 10.3389/fonc.2021.683608

**Published:** 2021-08-06

**Authors:** Yang Han, Yi Xuan, Xiaowen Liu, Hui Zhu, Meng Zhang, Dazhi Xu, Yanong Wang, Hong Cai

**Affiliations:** ^1^Department of Gastric Surgery, Fudan University Shanghai Cancer Center, Shanghai, China; ^2^Department of Oncology, Shanghai Medical College, Fudan University, Shanghai, China; ^3^Department of Radiology, Fudan University Shanghai Cancer Center, Shanghai, China; ^4^Department of Pathology, Fudan University Shanghai Cancer Center, Shanghai, China

**Keywords:** linitis plastica, quantitative definition, CT, tumor size, neoadjuvant therapy

## Abstract

Gastric linitis plastica (GLP) is a descriptive term but lacks a quantitative definition. Several relatively quantitative criteria had been proposed, such as tumor involving a limit of one-third or two-thirds of the gastric surface. However, these criteria needed doctors to subjectively judge tumor infiltration area, which made diagnosis difficult to be objective and reproducible. This study aimed to propose a quantitative diagnostic criterion for distinguishing GLP. We performed a retrospective cohort study of 2,907 patients with Borrmann III and IV gastric cancer (GC) who underwent gastrectomy between 2011 and 2018 in our center. The Kaplan–Meier curves showed that patients with an observed tumor size more than 8 cm had obviously lower overall survival (OS) and disease-free survival (DFS) rates than those with a size less than 8 cm(p < 0.001; p < 0.001). However, there was no significantly different prognosis of patients with tumor sizes between more than 8 cm and more than 10 cm (p = 0.248; p = 0.534). Moreover, patients with tumor sizes greater than 8 cm more presented with advanced stage and had extremely poor 3-year OS and DFS (31.4%; 29.3%), with a stronger propensity toward peritoneal metastasis. Therefore, we considered patients’ observed tumor size more than 8 cm as a critical value for distinguishing the prognosis of Borrmann III and IV GC. Furthermore, we proposed an observed tumor size more than 8 cm as a quantitative diagnostic criterion for GLP on the premise of satisfying the originally descriptive and pathological definition regardless of Borrmann type.

## Introduction

Gastric cancer (GC) is the fifth malignancy worldwide and the second most commonly diagnosed cancer in China (1, 2). Gastric linitis plastica (GLP) is a special phenotype of GC found in 7%–14% of cases and represents a particular entity (3). It is characterized macroscopically as a thickened stomach, with prominent diffusion of the tumor into the submucosal and muscular layers and microscopically by the association with signet ring cell features and diffuse and scirrhous histologic types (4, 5). GLP has a special predominance of distant lymph node metastasis, peritoneal metastasis, and ascites (6–8). As such, curative resection is possible in less than half of patients, and early recurrence is common, leading to a poor prognosis, median survival ranging from 6 to 12 months, and 5-year survival between 8% and 13% (9–13).

Despite these specific features, GLP still lacks a clear and standardized definition. GLP is used interchangeably with “Borrmann IV type carcinoma” and “scirrhous carcinoma” (12). However, these terms only include the partial characteristics of GLP and are often indiscriminately used to lead to confusion in the literature (6). The original definition of GLP is based on preoperative gastroscopy biopsies, CT scan, and postoperative surgical specimens. However, many GLP patients affected by advanced disease would not undergo gastrectomy, so that the typical definition based on postoperative surgical specimens would not always be possible. Moreover, the increasingly common practice of neoadjuvant chemotherapy or radiotherapy increases the need for preoperative diagnosis of GLP (14). In recent years, there has been an increasing development of liquid biopsy, defined as the preoperative sampling and analysis of GLP tissue (15). However, the repeatability and sensitivity of liquid biopsy are quite different. The concordance between fluid biopsy markers and clinical phenotypes is not satisfactory. Therefore, there is a need for a simple and specifically macroscopic criterion that could be used in clinical practice to aid surgeons and oncologists to arrive at a definite diagnosis preoperatively (16).

Several relatively quantitative criteria had been proposed in recent years. Pedrazzani et al. (11) defined GLP as a thickening and stiffening of the gastric wall that involved circumferentially at least one-third of the stomach. Then, Endo et al. (17) considered GLP as a gastric wall involving more than two-thirds of the stomach. Recently, Agnes et al. (6) proposed the definition as a thickening of the gastric wall that involved more than one-third of the gastric surface as a circumferential involvement of more than one area or a semicircular involvement of more than two areas. However, these definitions tended to be descriptive concepts and needed doctors to subjectively judge whether the tumor is more than one-third or two-thirds of the gastric surface by endoscope or CT scan, which made it difficult for surgeons and oncologists from different institutions to guarantee a uniform identification. Thus, the definition should be macroscopic, with a quantitatively critical value that the GLP phenotype is clearly identifiable preoperatively.

The Gastrointestinal Oncology Study Group of Japan Clinical Oncology Group (JCOG) grouped Borrmann IV with large Bormann III GC (≥8 cm in diameter) together in JCOG0210 and JCOG0501 due to the large Borrmann III with the same biological characteristics as Borrmann IV GC (18–20). In reference to Japanese studies, we proposed whether an observed tumor size ≥8 cm preoperatively by stomach enhanced CT scan could be used as a quantitative diagnosis for GLP on the premise of meeting the descriptive and pathological definition in China. The objective of this retrospective study was to propose a clearly quantitative diagnosis for GLP by survival analysis on the premise of satisfying the originally descriptive and pathological definition. Moreover, we explored clinicopathologic factors and evaluated the prognosis of GLP patients with or without neoadjuvant chemotherapy or radiotherapy. Our results would provide a firm foundation for the standardized and reproducible definition of GLP and help to define the best therapeutic options for it.

## Materials and Methods

### Patient Population

We retrospectively collected the records of 8,659 patients who underwent gastrectomy for GC between 2011 and 2018 in Fudan University Shanghai Cancer Center. All the records were reviewed by the same person (YH) to minimize missing data and control concordance. Information collected from medical records included age, sex, preoperative chemotherapy or chemoradiotherapy, surgical procedure, observed tumor size, pathological tumor size, pathologic stage, overall survival (OS), and disease-free survival (DFS). The study was conducted in accordance with the Declaration of Helsinki, and the protocol was approved by the ethics committee of Fudan University Shanghai Cancer Center (Project 1611166-2). The consent to participate was exempted in our study for the reason that this was a retrospective study only about clinical information.

### Observed Tumor Size

The observed tumor size is the maximum diameter of tumor measured preoperatively by stomach enhanced CT scan (21). The detailed methods are as follows. The stomach enhanced CT is performed using 64-section CT. Before CT examination, a patient should be prepared by overnight fasting or fasting for at least 6 h to empty the stomach. About 800–1,000 ml warm water is administered orally to distend the gastric lumen 10 min before the CT scan. The degree of gastric distension is considered to be adequate when the gastric lumen is distended greater than 50% of the expected maximal luminal distension. Our team estimates tumor size of GC with respect to the maximum long-axis diameter at the portal venous phase CT (60 s after the trigger threshold 100 HU on the abdominal aorta) (22). If there is no preoperative stomach enhanced CT scan in our center, we would make intraoperative tumor size instead of it based on the surgical records. Intraoperative tumor size is defined as the maximum diameter of tumor that is measured according to the JCGC criteria (23). Briefly, the resected stomach is scissored open along the greater curvature firstly so that the tumor lesions could be maintained intact. If the tumor is located at the greater curvature, the excised specimen would be cut open along the lesser curvature. The opened specimen is then affixed to a flat board, and the maximum diameter of tumor is measured and recorded. When tumor margin is unclear such as Borrmann IV GC, the resected stomach is then fixed by formalin for 1 h to make the margins clearer.

### Pathological Tumor Size

The pathological tumor size is the long-axis diameter of tumor according to the pathological report in our center. The detailed method of measurement for tumor is according to the JCGC criteria after the resected stomach has soaked in formalin overnight.

### Preoperative Therapy

According to the National Comprehensive Cancer Network (NCCN) guideline, advanced GC before radical surgery is generally recommended to be treated with either preoperative chemotherapy alone or preoperative induction chemotherapy followed by chemoradiation therapy. Preoperative chemotherapy is mostly that patients receive two or three cycles of S-1 and oxaliplatin (SOX, lasting 21 days) before surgery in our center. Chemoradiation is mostly that patients receive two cycles of SOX plus 45 Gy radiation administered concurrently with S-1 before surgery in our center (24).

### Statistical Analysis

All data and survival analyses were calculated using SPSS version 19.0 statistical software (SPSS, Chicago, USA). The clinical characteristics of patients were expressed as means with standard deviations. The significance of the covariate differences was determined using a two-tailed χ^2^ or Fisher’s exact test where appropriate. OS was calculated from the date of operation to the date of death or was censored at last follow-up. DFS was calculated from the date of operation to the first documented radiological recurrence or GC-related death. Survival curves were estimated with the Kaplan–Meier method and compared with the log-rank test. A Cox proportional hazards model was used to investigate the multivariate analysis and independent prognostic factors. All p-values <0.05 were considered to be statistically significant.

## Results

### Patients’ Characteristics

**Figure 1:**
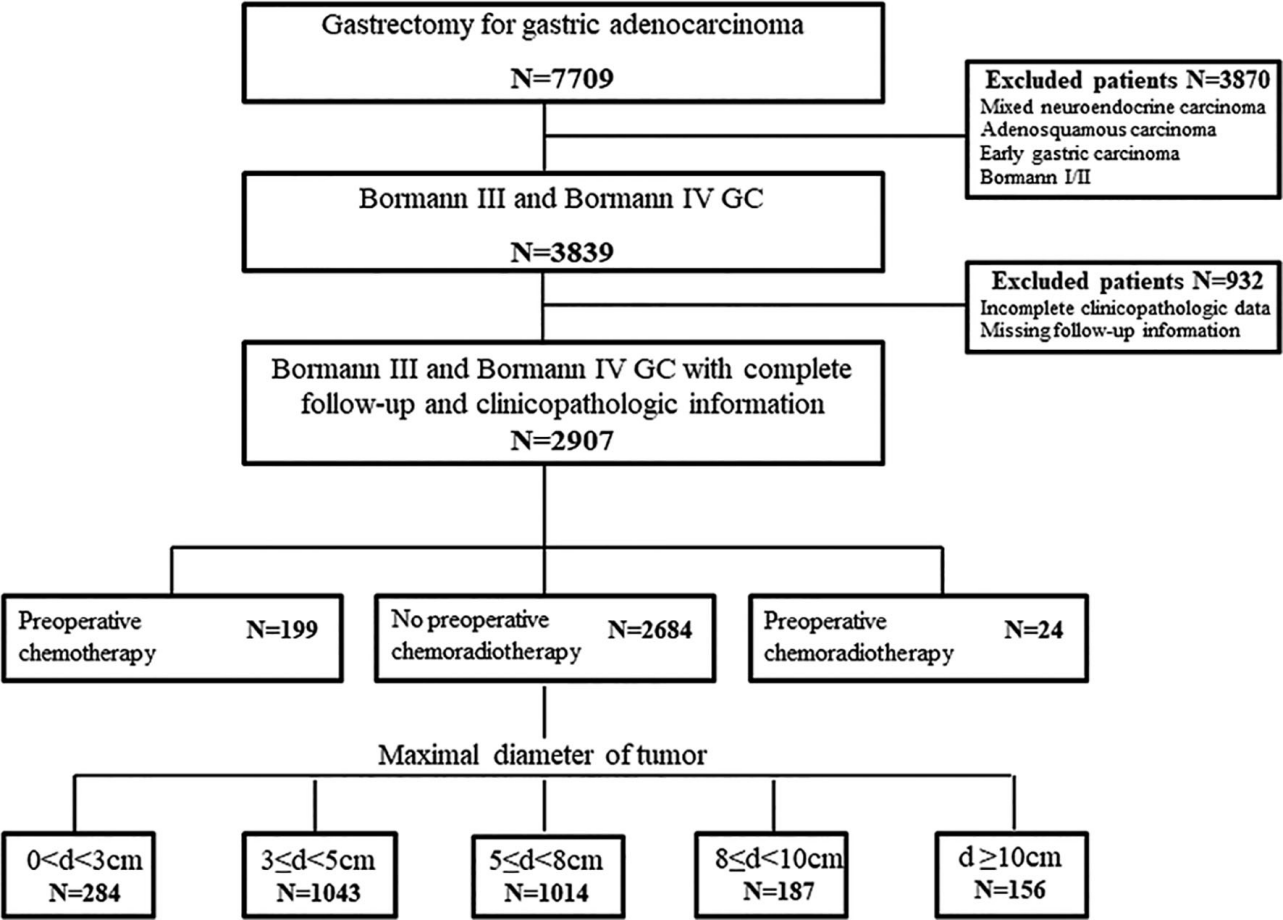
The flowchart of retrospective analysis for gastric linitis plastica (GLP).

The files of 7,709 patients who underwent gastrectomy for GC were reviewed. As the objective of this study was to propose a quantitative diagnosis for GLP by survival analysis, we only selected Borrmann III and Borrmann IV gastric adenocarcinoma as study population referring to JCOG0210 and JCOG0501. Among the 3,839 cases of Borrmann III and Borrmann IV, 932 records were not analyzable due to missing follow-up information or incompletely clinicopathologic data. In the remaining 2,907 patients, 199 cases with preoperative chemotherapy and 24 cases with preoperative chemoradiotherapy needed to consider the influence of chemotherapy or radiotherapy on prognosis. Therefore, a total of 2,684 patients were included in the analysis of quantitative diagnosis for GLP grouping by tumor size (**Figure 1**).

The general characteristics of the 2,684 GC were presented in **Table 1**. The group was composed of 2,382 Borrmann III and 302 Borrmann IV GC. The patient population of stages I, II, III, and IV were 264, 626, 1,745, and 49 cases, respectively. We classified the patients as five groups based on observed and pathological tumor diameter: d < 3 cm, 3 ≤d < 5 cm, 5 ≤ d < 8 cm, 8 ≤ d < 10 cm, d ≥ 10 cm. We were surprised to find that 11.9% (36/302) of patients’ pathological tumor sizes were less than 3 cm, and 25.8% (78/302) of patients’ sizes were between 3 and 5 cm among 302 Borrmann IV GC according to our pathological reports. This Borrmann IV GC obviously cannot be called GLP, which was why we have to do this study.

**Table 1:** The general characteristics, univariate and multivariate analysis of overall survival and disease-free survival of 2,684 GC patients.

		Overall survival				Disease-free survival			
	N	Univariate analysis		Multivariate analysis		Univariate analysis		Multivariate analysis	
	2,684	HR (95% CI)	p	HR (95% CI)	p	HR (95% CI)	p	HR (95% CI)	p
Gender			0.703				0.785		
Male	1,913	—	—			—	—		
Female	771	0.973 (0.846–1.120)	0.703			0.981 (0.852–1.128)	0.785		
Age			<0.001*		<0.001*		<0.001*		<0.001*
<45 years	239	—	—	—	—	—	—	—	—
45 ≤ years < 65	1,520	1.271 (0.988–1.637)	0.062	1.287 (0.999–1.659)	0.051	1.236 (0.961–1.592)	0.099	1.233 (0.956–1.591)	0.107
≥65 years	925	1.675 (1.294–2.168)	<0.001*	1.711 (1.319–2.219)	<0.001*	1.621 (1.252–2.098)	<0.001*	1.582 (1.217–2.058)	0.001*
Location			<0.001*				<0.001*		0.030*
EGJ	573	—	—			—	—	—	—
Gastric fundus	25	1.424 (0.797–2.544)	0.233			1.521 (0.851–2.719)	0.157	1.400 (0.775–2.530)	0.265
Gastric corpus	729	1.021 (0.858–1.216)	0.812			0.985 (0.827–1.172)	0.863	0.878 (0.733–1.052)	0.158
Gastric angle	244	0.573 (0.430–0.764)	<0.001*			0.545 (0.409–0.727)	<0.001*	0.720 (0.537–0.965)	0.028
Gastric antrum	867	0.774 (0.650–0.921)	0.004*			0.742 (0.624–0.884)	0.001*	0.777 (0.651–0.929)	0.005*
over one area	246	1.379 (1.107–1.718)	0.004*			1.365 (1.096–1.700)	0.006*	0.898 (0.709–1.136)	0.369
Borrmann type			<0.001*				<0.001*		
III	2,382	—	—			—	—		
IV	302	1.466 (1.219–1.764)	<0.001*			1.475 (1.226–1.775)	<0.001*		
Observed size			<0.001*		<0.001*		<0.001*		<0.001*
<3 cm	284	—	—	—	—	—	—	—	—
3 ≤ d < 5 cm	1,043	1.547 (1.170–2.045)	0.002*	1.198 (0.900–1.594)	0.215	1.535 (1.161–2.029)	0.003*	1.091 (0.816–1.458)	0.558
5 ≤ d < 8 cm	1,014	2.314 (1.758–3.046)	<0.001*	1.425 (1.068–1.902)	0.016	2.308 (1.753–3.038)	<0.001*	1.300 (0.968–1.747)	0.081
8 ≤ d < 10 cm	187	4.043 (2.912–5.613)	<0.001*	2.203 (1.564–3.103)	<0.001*	3.959 (2.852–5.497)	<0.001*	1.972 (1.389–2.802)	<0.001*
≥10 cm	156	4.670 (3.332–6.547)	<0.001*	2.387 (1.677–3.398)	<0.001*	4.230 (3.019–5.928)	<0.001*	1.924 (1.333–2.777)	<0.001*
Pathology size			<0.001*				<0.001*		
<3 cm	593	—	—			—	—		
3 ≤ d < 5 cm	1,096	1.514 (1.252–1.829)	<0.001*			1.502 (1.243–1.816)	<0.001*		
5 ≤ d < 8 cm	737	1.980 (1.628–2.409)	<0.001*			1.982 (1.629–2.411)	<0.001*		
8 ≤ d < 10 cm	155	2.553 (1.935–3.369)	<0.001*			2.534 (1.921–3.344)	<0.001*		
≥10 cm	103	4.019 (2.965–5.447)	<0.001*			3.674 (2.712–4.977)	<0.001*		
T stage			<0.001*		<0.001*		<0.001*		<0.001*
T1	148	—	—	—	—	—	—	—	—
T2	343	1.225 (0.792–1.896)	0.362	1.041 (0.670–1.617)	0.859	1.196 (0.773–1.851)	0.422	0.913 (0.575–1.449)	0.698
T3	689	1.907 (1.280–2.842)	0.002*	1.159 (0.766–1.753)	0.486	1.807 (1.213–2.693)	0.004*	0.793 (0.479–1.314)	0.368
T4	1,504	3.095 (2.121–4.516)	<0.001*	1.637 (1.100–2.436)	0.015*	3.147 (2.156–4.592)	<0.001*	1.181 (0.713–1.958)	0.518
N stage			<0.001*		<0.001*		<0.001*		<0.001*
N0	646	—	—	—	—	—	—	—	—
N1	489	1.271 (1.000–1.615)	0.050	1.172 (0.920–1.494)	0.199	1.283 (1.010–1.630)	0.041*	0.990 (0.745–1.316)	0.946
N2	608	1.860 (1.500–2.305)	<0.001*	1.585 (1.272–1.976)	<0.001*	1.895 (1.528–2.349)	<0.001*	1.282 (0.951–1.729)	0.103
N3	941	3.606 (2.982–4.361)	<0.001*	2.809 (2.299–3.434)	<0.001*	3.685 (3.046–4.456)	<0.001*	2.286 (1.706–3.065)	<0.001*
M stage			0.001*				<0.001*		
M0	2,635	—	—	—	—	—	—		
M1	49	2.024 (1.381–2.968)	0.001*	1.624 (1.105–2.386)	0.014*	2.701 (1.842–3.961)	<0.001*		
AJCC stage			<0.001*				<0.001*		<0.001*
I	264	—	—			—	—	—	—
II	626	1.756 (1.216–2.537)	0.003*			1.751 (1.212–2.529)	0.003*	1.478 (0.922–2.370)	0.105
III	1,745	4.190 (2.999–5.854)	<0.001*			4.312 (3.087–6.025)	<0.001*	1.830 (1.019–3.288)	0.043
IV	49	6.471 (3.927–10.663)	<0.001*			8.818 (5.350–14.535)	<0.001*	3.736 (1.896–7.365)	<0.001*
Differentiation			<0.001*				<0.001*		
High	11	—	—			—	—		
Moderate	442	0.682 (0.252–1.845)	0.451			0.747 (0.276–2.021)	0.565		
Low	1,352	1.120 (0.419–2.994)	0.822			1.216 (0.455–3.252)	0.697		
High-moderate	25	0.469 (0.126–1.746)	0.259			0.490 (0.131–1.824)	0.287		
Moderate-low	757	0.901 (0.335–2.418)	0.836			0.970 (0.361–2.603)	0.951		
Unreported	97	1.239 (0.447–3.436)	0.681			1.320 (0.476–3.661)	0.594		

AJCC, American Joint Committee on Cancer; CI, confidence interval; GC, gastric cancer; HR, hazard ratio.

*p < 0.05 indicated that the 95% CI of HR was not including 1.

Moreover, Cox univariate analysis suggested that the decreased OS and DFS were associated with age, tumor location, observed and pathological tumor size, pT stage, pN stage, pM stage, American Joint Committee on Cancer (AJCC) stage, and differentiation. Multivariate analysis confirmed that age, observed tumor size, pT stage, pN stage, and pM stage remained as independent prognostic factors in Borrmann III and IV GC, not including pathological tumor size (**Table 1**).

**Figure 2:**
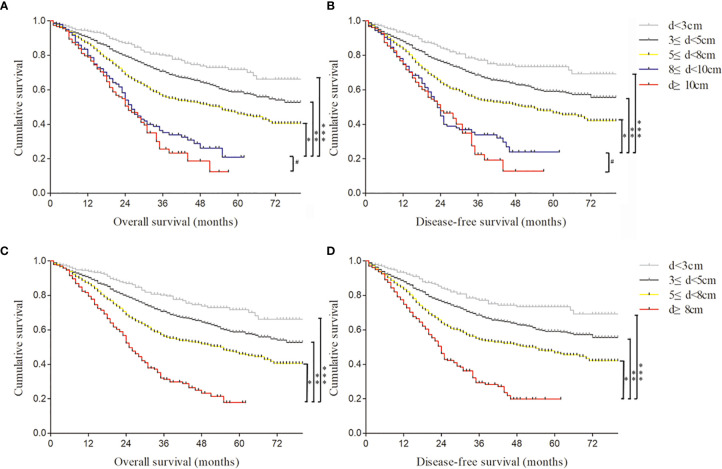
The Kaplan–Meier curves were used to estimate different survival rates of 2,684 gastric cancer (GC) specimens grouped by observed tumor size. **(A)** The overall survival of 2,684 GC specimens divided into five groups according to the observed tumor size. *, **, ***log rank p < 0.001; #log rank p = 0.248. **(B)** The disease-free survival of 2,684 GC specimens divided into five groups according to the observed tumor size. *, **, ***log rank p < 0.001; #log rank p = 0.534. **(C)** The overall survival of 2,684 GC specimens divided into four groups according to the observed tumor size. *, **, ***log rank p < 0.001. **(D)** The disease-free survival of 2,684 GC specimens divided into four groups according to the observed tumor size. *, **, ***log rank p < 0.001.

Thus, Kaplan–Meier curves were used to estimate the survival of 2,684 GC specimens grouped by observed tumor size according to the Cox analysis results (**Figure 2**). It was shown that patients with an observed tumor size more than 8 cm had obviously lower OS and DFS rates than those with size less than 8 cm(p < 0.001; p < 0.001). However, there was no significantly different prognosis of patients with observed tumor sizes between more than 8 cm and more than 10 cm (p = 0.248; p = 0.534) (**Figures 2A, B**). Furthermore, to remove the influence of the tumor stage on prognosis, the patients were stratified based on the AJCC stage to analyze Kaplan–Meier curves (**Figure S1**). The results showed that the group of patients with more than 8-cm tumor had worse OS and DFS rates than that with less than 8 cm at stage III disease (p < 0.001; p < 0.001) (**Figure S1 C1, 2**). In other stages, there was no statistical difference in survival curves among different size groups due to the small sample of tumor size with more than 8 cm, such as 3/263 at stage I, 44/626 at stage II, and 9/49 at stage IV (**Figure S1 A, B, D**). Therefore, patients’ observed tumor size more than 8 cm was a critical value for distinguishing prognosis of Borrmann III and IV GC based on survival analysis. Furthermore, we proposed a preoperatively observed tumor size larger than 8 cm as a quantitative diagnostic criterion of GLP on the premise of satisfying the originally descriptive and pathological definition regardless of Borrmann III or Borrmann IV type.

### Gastric Linitis Plastica Characteristics According to Our Criteria

**Figure 3:**
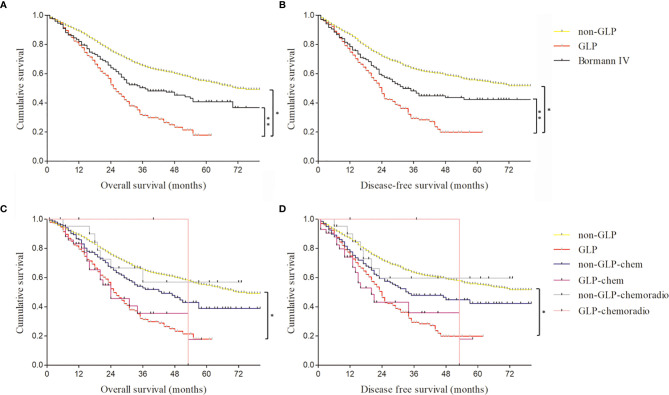
The comparison of survival curves of gastric linitis plastica (GLP) and Borrmann IV patients with or without neoadjuvant chemotherapy and chemoradiotherapy. **(A)** The overall survival of GLP, non-GLP, and Borrmann IV patients in our study. *log rank p < 0.001; **log rank p = 0.015. **(B)** The disease-free survival of GLP, non-GLP, and Borrmann IV patients in our study. *log rank p < 0.001; **log rank p = 0.012. **(C)** The overall survival of GLP and non-GLP patients with or without neoadjuvant chemotherapy and chemoradiotherapy. *log rank p < 0.001. **(D)** The disease-free survival of GLP and non-GLP patients with or without neoadjuvant chemotherapy and chemoradiotherapy. *log rank p < 0.001.

According to our quantitative standards, of the 2,684 patients in our study, 343 (12.8%) met our quantitatively diagnostic criteria of GLP. Among 343 GLP patients, we found that Borrmann III GC was in the majority, accounting for 69.7% (239/343), rather than Borrmann IV type (**Figure S2**). The age of GLP varied from 22 and 84, with a median age of 59, and the male-to-female ratio was 2.3:1 (**Table 2**). The median OS of GLP after radical gastrectomy was 20 months, and the median DFS of GLP was 18 months. The 3- and 5-year OS rates were 31.4% and 17.9%, respectively, and the 3- and 5-year DFS rates were 29.3% and 19.8%, respectively, in the GLP group (**Figures 3A, B**). However, in the non-GLP group, 3- and 5-year OS rates were 65.6% and 54.8%, respectively, and the 3- and 5-year DFS rates were 63.3% and 55.2%, respectively. GLP had a significantly shorter OS and DFS than did those without GLP (p < 0.001; p < 0.001) (**Figures 3A, B**).

**Table 2:** The difference of the clinicopathological features between GLP and non-GLP.

	GLP	Non-GLP	P
	N = 343	N = 2341	
Age (mean ± SD)	59.58 ± 10.85	59.59 ± 10.74	0.771
Gender			0.407
Male	238 (69.4%)	1,675 (71.6%)	
Female	105 (30.6%)	666 (28.4%)	
Invasion adjacent organs			<0.001*
Pancreas	15 (4.4%)	38 (1.6%)	
Transverse colon	10 (2.9%)	17 (0.7%)	
Peritoneal metastasis	22 (6.4%)	25 (1.1%)	
Pelvic cavity	0 (0.0%)	4 (0.2%)	
Type of gastrectomy			<0.001*
Proximal	12 (3.5%)	348 (14.9%)	
Distal	64 (18.7%)	1,157 (49.4%)	
Total	267 (77.8%)	833 (35.6%)	
Lymph node dissection			<0.001*
D0/D1	9 (2.6%)	93 (4.0%)	
D2/D3	334 (97.4%)	2,248 (96.0%)	
Resection			0.176
R0	338 (98.5%)	2,325 (99.3%)	
R1/R2	5 (1.5%)	16 (0.7%)	
Pathological type			<0.001*
Mucinous adenocarcinoma	25 (7.3%)	139 (5.9%)	
Signet-ring cell	124 (36.2%)	518 (22.1%)	
Undifferentiated	0 (0.0%)	2 (0.1%)	
Bormann type			<0.001*
Borrmann III	239 (69.7%)	2,143 (91.5%)	
Borrmann IV	104 (30.3%)	198 (8.5%)	
pT stage			<0.001*
pT1/T2	9 (2.6%)	482 (20.6%)	
pT3/T4	334 (97.4%)	1,859 (79.4%)	
pN stage			<0.001*
pN0/N1	69 (20.1%)	1,066 (45.5%)	
pN2/N3	274 (79.9%)	1,275 (54.5%)	
peritoneal lavage cytology			<0.001*
Negative	234 (92.9%)	2,203 (99.1%)	
Positive	18 (7.1%)	21 (0.9%)	
Tumor thrombus in vessel			<0.001*
Negative	100 (29.2%)	1,001 (42.8%)	
Positive	243 (70.8%)	1,340 (57.2%)	
Tumor thrombus in lymph			<0.001*
Negative	46 (13.4%)	613 (26.2%)	
Positive	297 (86.6%)	1,728 (73.8%)	
Tumor invasion in nerve			<0.001*
Negative	78 (22.7%)	894 (38.2%)	
Positive	265 (77.3%)	1,447 (61.8%)	
Differentiation			<0.001*
Moderate	29 (8.5%)	413 (17.6%)	
Moderate-low	77 (22.4%)	680 (29.0%)	
Low	222 (64.7%)	1,130 (48.3%)	

p-values are based on chi-square or Fisher’s exact test.

*Significant difference.

GLP, gastric linitis plastica.

The comparative analysis of the clinical characteristics of the GLP and non-GLP specimens was presented in **Table 2**. GLP patients had more total gastrectomy (77.8% *vs*. 35.6%) and had signet ring cell (36.2% *vs*. 22.1%) and poorly differentiated histologic types (64.7% *vs*. 48.3%) than those with non-GLP (**Table 2**). Peritoneal metastasis and positive peritoneal lavage cytology were more frequent in the GLP group than in the non-GLP group (6.4% *vs*. 1.1% and 7.1% *vs*. 0.9%, respectively). The proportion of pT3+4 stage and pN2+3 stage in the GLP group was obviously higher than that in the non-GLP (97.4% *vs*. 79.4% and 79.9% *vs*. 54.5%, respectively). GLP patients were more frequently with positive tumor thrombus in vessel and lymph and tumor invasion in nerve than non-GLP patients (70.8% *vs*. 57.2%, 86.6% *vs*. 73.8%, and 77.3% *vs*. 61.8%, respectively).

Furthermore, we compared the survival rates and prognoses of GLP patients with or without chemotherapy and chemoradiotherapy (**Figures 3C, D**). The results showed there was no significant survival benefit after radiotherapy or chemotherapy, which may be related to the small sample size of preoperative chemotherapy and chemoradiotherapy patients. The difference of outcome of prognosis evaluation between surgery and neoadjuvant chemotherapy or chemoradiotherapy needs large-scale randomized controlled clinical trials for further verification.

### The Comparison of Clinicopathological Characteristics Between Borrmann IV and Gastric Linitis Plastica According to Our Criteria

There were only 302 Borrmann IV GC patients among the 2,684 specimens according to the pathological reports. The 3- and 5-year OS rates of Borrmann IV GC patients were 50.4% and 40.8%, respectively, and the 3- and 5-year DFS rates were 47.8% and 42%, respectively (**Figures 3A, B** and **Table 3**). Compared with GLP according to our criteria, Borrmann IV patients in our study had significantly better OS and DFS. Moreover, the proportion of total gastrectomy, pT3+4 stage, and pN2+3 stage in the Borrmann IV group was obviously lower than that in the GLP according to our criteria, all of which were at variance with the classical theories about linitis plastica (**Table 3**).

**Table 3:** The difference of the clinicopathological features between GLP and Borrmann IV GC.

	GLP	Borrmann IV	p
	N = 343	N = 302	
OS			0.015*
3-year	0.314	0.504	
5-year	0.179	0.408	
DFS			0.012*
3-year	0.293	0.478	
5-year	0.198	0.420	
Invasion adjacent organs			0.189
Pancreas	15 (4.4%)	14 (4.6%)	
Transverse colon	10 (2.9%)	3 (1%)	
Peritoneal metastasis	22 (6.4%)	9 (3%)	
Pelvic cavity	0 (0.0%)	0 (0%)	
Type of gastrectomy			0.001*
Proximal	12 (3.5%)	27 (8.9%)	
Distal	64 (18.7%)	94 (31.1%)	
Total	267 (77.8%)	181 (59.9%)	
Lymph node dissection			0.501
D0/D1	9 (2.6%)	11 (3.6%)	
D2/D3	334 (97.4%)	291 (96.4%)	
Resection			1
R0	338 (98.5%)	298 (98.7%)	
R1/R2	5 (1.5%)	4 (1.3%)	
Pathological type			0.132
Mucinous adenocarcinoma	25 (7.3%)	16 (5.3%)	
Signet-ring cell	124 (36.2%)	135 (44.7%)	
Undifferentiated	0 (0.0%)	0 (0%)	
Borrmann type			0.001*
Borrmann III	239 (69.7%)	0 (0%)	
Borrmann IV	104 (30.3%)	302 (100%)	
pT stage			0.001*
pT1/T2	9 (2.6%)	32 (10.6%)	
pT3/T4	334 (97.4%)	270 (89.4%)	
pN stage			0.003*
pN0/N1	69 (20.1%)	92 (30.5%)	
pN2/N3	274 (79.9%)	210 (69.5%)	
Peritoneal lavage cytology			0.112
Negative	234 (92.9%)	240 (96.4%)	
Positive	18 (7.1%)	9 (3.6%)	
Tumor thrombus in vessel			0.306
Negative	100 (29.2%)	100 (33.1%)	
Positive	243 (70.8%)	202 (66.9%)	
Tumor thrombus in lymph			0.501
Negative	46 (13.4%)	47 (15.6%)	
Positive	297 (86.6%)	255 (84.4%)	
Tumor invasion in nerve			0.460
Negative	78 (22.7%)	77 (25.5%)	
Positive	265 (77.3%)	225 (74.5%)	
Differentiation			0.007*
Moderate	29 (8.5%)	20 (6.6%)	
Moderate-low	77 (22.4%)	40 (13.2%)	
Low	222 (64.7%)	221 (73.2%)	

p-values are based on chi-square or Fisher’s exact test.

*Significant difference.

DFS, disease-free survival; GC, gastric cancer; GLP, gastric linitis plastica; OS, overall survival.

## Discussion

GLP is a long-known term that might date back to the 16th and 17th centuries (25). It was defined until 1947 by Arthur Stout (26) as a specific type of gastric carcinoma characterized macroscopically by a major segmental or diffuse thickening of the gastric wall and microscopically by the existence of poorly cohesive and/or signet ring cells. However, this definition tended to be a descriptive concept, missing detailed quantitative standards. Although in the following year, several relatively quantitative criteria were proposed. For example, Nakamura defined typical GLP as the involvement of more than one-fourth of the stomach (27), Pedrazzani et al. (11) proposed a critical value as one-third thickening and stiffening of the stomach (11), and Endo et al. (17) considered GLP as gastric wall involving a limit of two-thirds of the stomach. However, neither of these classifications was an accepted standard. These criteria needed doctors to subjectively judge whether the tumor was more than one-third or two-thirds of the gastric surface by endoscope or CT scan, which made these definitions difficult to be objective and reproducible.

Besides, GLP was interchangeably but not accurately termed “Borrmann IV type carcinoma,” “scirrhous carcinoma,” “Lauren carcinoma,” or “signet cell carcinoma” (28). In Japan, the term “scirrhous gastric cancer,” which commonly grouped Borrmann IV with large Borrmann III (≥8 cm in diameter) GC together, was often, but inconsistently, used confusedly with GLP to describe this phenotype of GC (18, 19, 29). Therefore, in our study, we explored whether an observed tumor size larger than 8 cm was used as a quantitative standard for GLP on the premise of satisfying the originally descriptive and pathological definition.

A total of 2,684 Borrmann III and Borrmann IV GC patients without preoperative chemotherapy or radiochemotherapy from the 7,709 GC database in our department were included in the analysis grouped by observed tumor size. It was shown that patients’ observed tumor size of more than 8 cm was a critical value for distinguishing prognosis from different tumor sizes based on survival analysis. Moreover, we proposed a preoperative observed tumor size larger than 8 cm by CT scan as a quantitatively diagnostic criterion of GLP on the prerequisite of meeting the requirement of originally descriptive and pathological definition. According to our quantitative standard, of the 2,684 patients in our study, 343 (12.8%) met the diagnostic criteria of GLP. GLP patients presented with more advanced stage and had extremely poor 3-year survival. More of these patients underwent total gastrectomy, with a stronger propensity toward peritoneal metastasis. These clinical characters of GLP according to our quantitative definition were consistent with previously classical theory and literature. But the results about median age and male-to-female ratio in our study did not show incidence characteristics such as younger age at diagnosis and female predominance as reported previously (3, 30). The reason for that perhaps was our sample selection bias. The tumor stage of these GLP patients enrolled in our study was relatively early. Those GLP patients with definite peritoneal metastasis or poor physical condition who had no opportunity of surgery generally would not be admitted to hospital in our department.

Borrmann classification was based on the macroscopically endoscopic/endoluminal aspect of the tumor, which was a subjective judgment, especially for Borrmann type IV (31). Borrmann IV GC was described as diffuse and infiltrative characteristics often lacking clear demarcation of the tumor edge (23). Borrmann IV and large Borrmann III (≥8 cm in diameter) GC were grouped in JCOG0210 and JCOG0501 due to the large Borrmann III with the same biological characteristics as Borrmann IV GC (18, 19). However, the patients with Borrmann IV tumors localized in less than two-thirds of the stomach were reported to have similar survival as patients with other non-scirrhous GC (17), which indicates that definitions based exclusively on the Borrmann classification underrepresent GLP. In our study, of 302 Borrmann IV GC, 11.9% (36/302) patients’ observed tumor sizes were less than 3 cm and 47.7% (114/302) patients’ sizes were less than 5 cm. The 3- and 5-year OS rates of Borrmann IV GC patients were 50.4% and 40.8%, respectively, and the 3- and 5-year DFS rates were 47.8% and 42%, which were inconsistent with the classical theory about linitis plastica. Therefore, not all of the Borrmann IV GC could be defined as GLP. Our team set an observed tumor size larger than 8 cm as a quantitative diagnostic criterion for GLP regardless of Borrmann type.

In addition, we took the observed tumor size preoperatively as a supplementary diagnostic standard for GLP, not pathology tumor size postoperatively in our study. It was observed that tumor size, not pathology tumor size, was an independent predictor of prognosis for Borrmann III and IV GC. Moreover, there is the need for a preoperatively quantitative critical value that the GLP phenotype is clearly identifiable, not postoperative one. The observed tumor size by stomach enhanced CT scan would guarantee a relative uniform identification of GLP and could be simply used in clinical practice preoperatively for oncologists and surgeons from different institutions (16, 21).

Moreover, the optimum treatment strategy for GLP is unknown (32). It is not clear whether patients with GLP could gain benefit from neoadjuvant chemotherapy or chemoradiation (33). Our results suggested that neoadjuvant chemotherapy or chemoradiation for GLP followed by gastrectomy did not bring obvious survival benefits. But our study sample size was small, the follow-up time was short, and the statistical error made our negative clinical curative effect need further research. The JCOG0501 trial, a phase III study of neoadjuvant chemotherapy with S-1/cisplatin in scirrhous type GC, showed that the addition of neoadjuvant chemotherapy did not appear to affect the survival rate of the scirrhous type GC patients and was not recommended (18). However, 80% of the patients enrolled in the group were N0/N1 stage in the JCOG0501 study. The high proportion of the early stage probably resulted in a negative conclusion. It was worth rethinking whether neoadjuvant chemotherapy could benefit the survival of patients with locally advanced GLP, especially in China. Therefore, more high-level evidence-based medical studies are expected to evaluate the value of neoadjuvant therapy of GLP.

Nevertheless, our study had some limitations. The major limitation of this retrospective study was the selection bias of samples source. Our department is gastric surgery, where admitted patients could undergo surgical treatment. Therefore, those GLP patients with tumor distant metastasis or poor physical condition would be refused admission to our outpatient department. Even if GLP patients are hospitalized in our department, these patients generally have no accompanying ascites or peritoneal metastasis preoperative, which leads to the tumor stage of GLP enrolled in our study relatively early. Moreover, the data were collected retrospectively with a limited number of patients in our single-center study, not multicenter. Therefore, there is a need for large-scale clinical validation.

## Conclusion

In summary, we considered patients’ observed tumor size of more than 8 cm by stomach enhanced CT as a critical value for distinguishing the prognosis of Borrmann III and IV GC. We proposed a preoperatively observed tumor size larger than 8 cm as a supplementary quantitative diagnosis for GLP on the premise of satisfying the originally descriptive and pathological definition. Moreover, neoadjuvant chemotherapy or chemoradiation for GLP patients followed by gastrectomy did not bring obvious survival benefits. In a word, this was a preliminary conclusion in a single-center study, which required us to enlarge the sample size to verify it. Next, we will focus on evaluating the value of neoadjuvant therapy of GLP in future studies.

## Data Availability Statement

The original contributions presented in the study are included in the article/**Supplementary Material**. Further inquiries can be directed to the corresponding author.

## Ethics Statement 

Written informed consent was obtained from the individual(s) for the publication of any potentially identifiable images or data included in this article.

## Author Contributions

YH and HC conceived and designed the study and helped draft the manuscript. YX, XL, HZ, and MZ performed all data collection and conducted the statistical analysis. YW and DX revised the manuscript. All authors contributed to the article and approved the submitted version.

## Funding

This study was funded by a grant from the National Natural Science Foundation of China (No. 81802439).

## Conflict of Interest

The authors declare that the research was conducted in the absence of any commercial or financial relationships that could be construed as a potential conflict of interest.

## Publisher’s Note

All claims expressed in this article are solely those of the authors and do not necessarily represent those of their affiliated organizations, or those of the publisher, the editors and the reviewers. Any product that may be evaluated in this article, or claim that may be made by its manufacturer, is not guaranteed or endorsed by the publisher.

## Supplementary Material

Supplementary Figure 1 The survival curves of 2684 GC specimens grouped by observed tumor size stratified based on the AJCC stage. **(A1)** The overall survival of AJCC I stage GC specimens divided into 4 groups according to observed tumor size. **(A2)** The disease-free survival of AJCC I stage GC specimens divided into 4 groups according to observed tumor size. **(B1)** The overall survival of AJCC II stage GC specimens divided into 4 groups according to observed tumor size. *log rank P=0.035. **(B2)** The disease-free survival of AJCC II stage GC specimens divided into 4 groups according to observed tumor size. *log rank P=0.029. **(C1)** The overall survival of AJCC III stage GC specimens divided into 4 groups according to observed tumor size. *,**,***log rank P<0.001. **(C2)** The disease-free survival of AJCC III stage GC specimens divided into 4 groups according to observed tumor size. *,**,***log rank P<0.001. **(D1)** The overall survival of AJCC IV stage GC specimens divided into 4 groups according to observed tumor size. **(D2)** The disease-free survival of AJCC IV stage GC specimens divided into 4 groups according to observed tumor size.

Supplementary Figure 2 The diagram of GLP composition by Bormann III and IV GC according to our quantitative standard.

Supplementary Figure 3 Receiver operating characteristic (ROC) curves of stratification analysis by tumor size as Bormanns IV type GC as control. **(A1)** The ROC curve of tumor size less than 3cm for the prediction of overall survival. **(A2)** The ROC curve of tumor size less than 3cm for the prediction of disease-free survival. **(B1)** The ROC curve of tumor size between 3cm and 5cm for the prediction of overall survival. **(B2)** The ROC curve of tumor size between 3cm and 5cm for the prediction of disease-free survival. **(C1)** The ROC curve of tumor size between 5cm and 8cm for the prediction of overall survival. **(C2)** The ROC curve of tumor size between 5cm and 8cm for the prediction of disease-free survival. **(D1)** The ROC curve of tumor size more than 8cm for the prediction of overall survival. **(D2)** The ROC curve of tumor size more than 8cm for the prediction of disease-free survival.

Supplementary Figure 4 Receiver operating characteristic (ROC) curve for tumor size. *The optimal cut-off point of tumor size was 5.250cm.
