# Supplementary figures and images for: Development of a Quantitative Diagnostic Criterion for Gastric Linitis Plastica: Findings From a Large Single-Institutional Study
Source: Front Oncol. 2021 Aug 6;11:683608. doi: 10.3389/fonc.2021.683608 (PMC8377468; doi:10.3389/fonc.2021.683608)

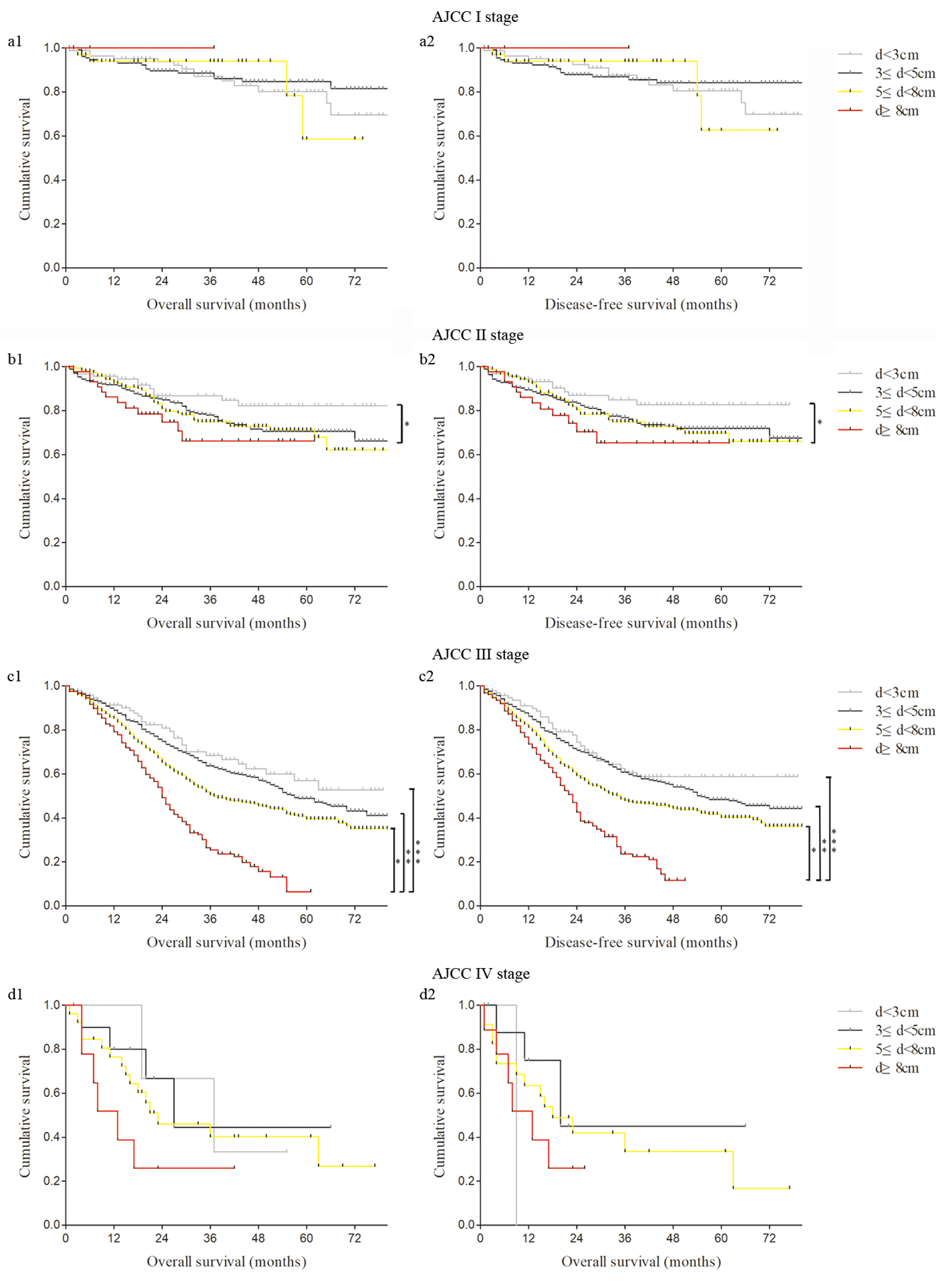

Supplement: Supplementary Figure 1 — The survival curves of 2684 GC specimens grouped by observed tumor size stratified based on the AJCC stage. (A1) The overall survival of AJCC I stage GC specimens divided into 4 groups according to observed tumor size. (A2) The disease-free survival of AJCC I stage GC specimens divided into 4 groups according to observed tumor size. (B1) The overall survival of AJCC II stage GC specimens divided into 4 groups according to observed tumor size. *log rank P=0.035. (B2) The disease-free survival of AJCC II stage GC specimens divided into 4 groups according to observed tumor size. *log rank P=0.029. (C1) The overall survival of AJCC III stage GC specimens divided into 4 groups according to observed tumor size. *,**,***log rank P<0.001. (C2) The disease-free survival of AJCC III stage GC specimens divided into 4 groups according to observed tumor size. *,**,***log rank P<0.001. (D1) The overall survival of AJCC IV stage GC specimens divided into 4 groups according to observed tumor size. (D2) The disease-free survival of AJCC IV stage GC specimens divided into 4 groups according to observed tumor size. [file Image_1.tif]

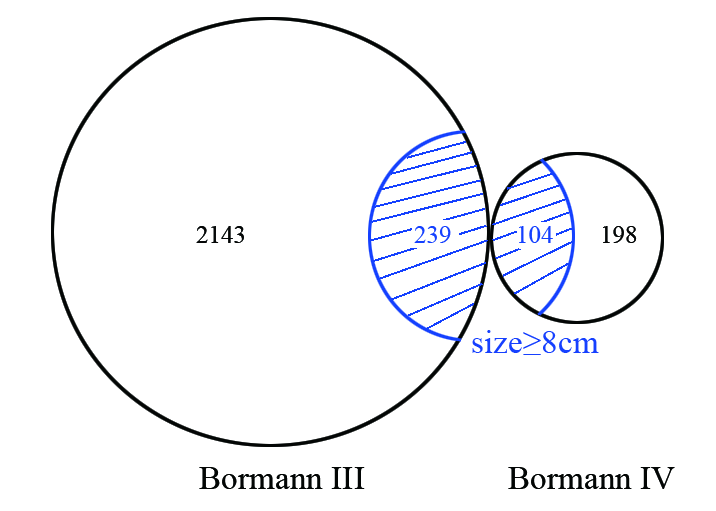

Supplement: Supplementary Figure 2 — The diagram of GLP composition by Bormann III and IV GC according to our quantitative standard. [file Image_2.tif]

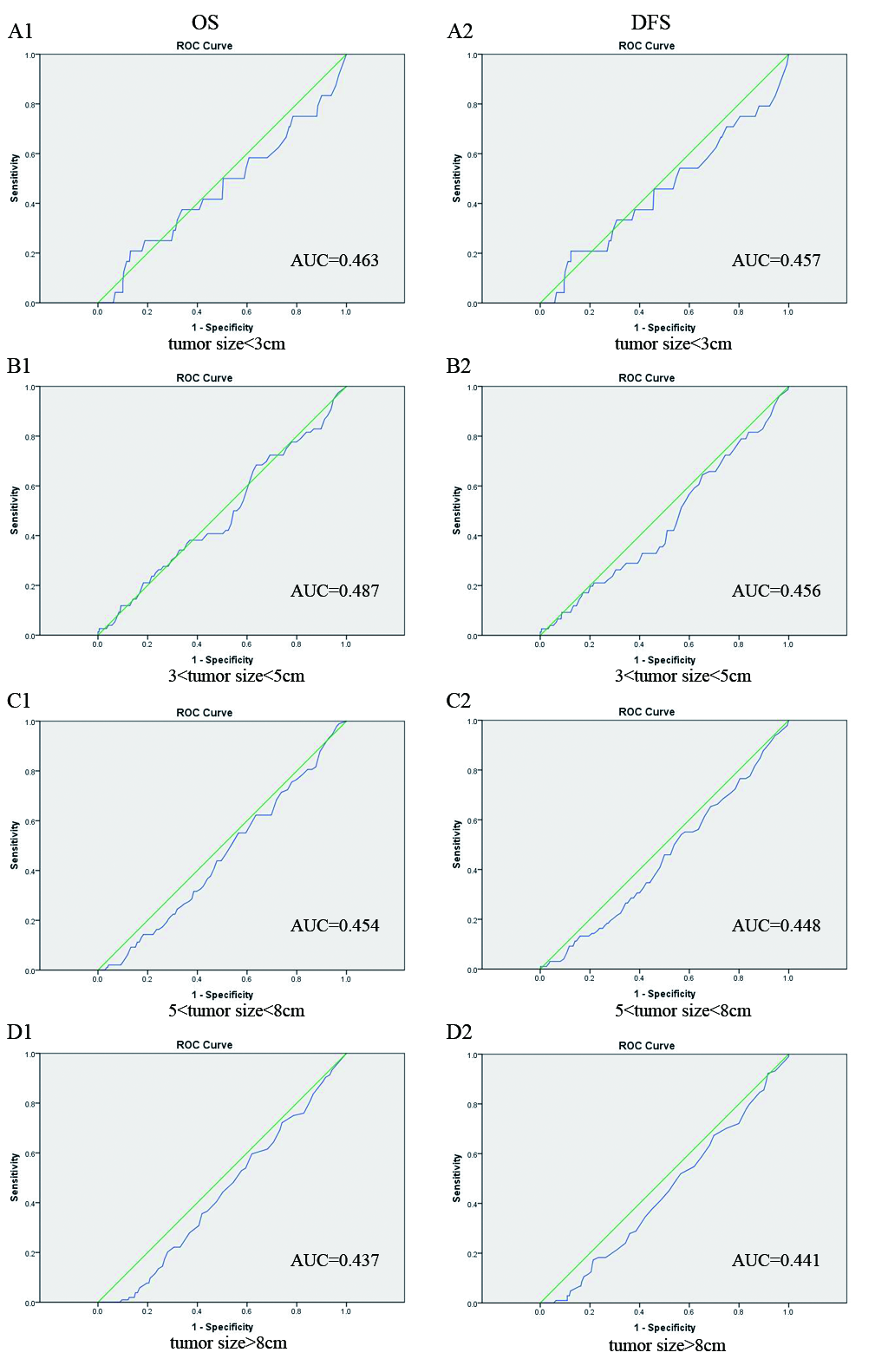

Supplement: Supplementary Figure 3 — Receiver operating characteristic (ROC) curves of stratification analysis by tumor size as Bormanns IV type GC as control. (A1) The ROC curve of tumor size less than 3cm for the prediction of overall survival. (A2) The ROC curve of tumor size less than 3cm for the prediction of disease-free survival. (B1) The ROC curve of tumor size between 3cm and 5cm for the prediction of overall survival. (B2) The ROC curve of tumor size between 3cm and 5cm for the prediction of disease-free survival. (C1) The ROC curve of tumor size between 5cm and 8cm for the prediction of overall survival. (C2) The ROC curve of tumor size between 5cm and 8cm for the prediction of disease-free survival. (D1) The ROC curve of tumor size more than 8cm for the prediction of overall survival. (D2) The ROC curve of tumor size more than 8cm for the prediction of disease-free survival. [file Image_3.tif]

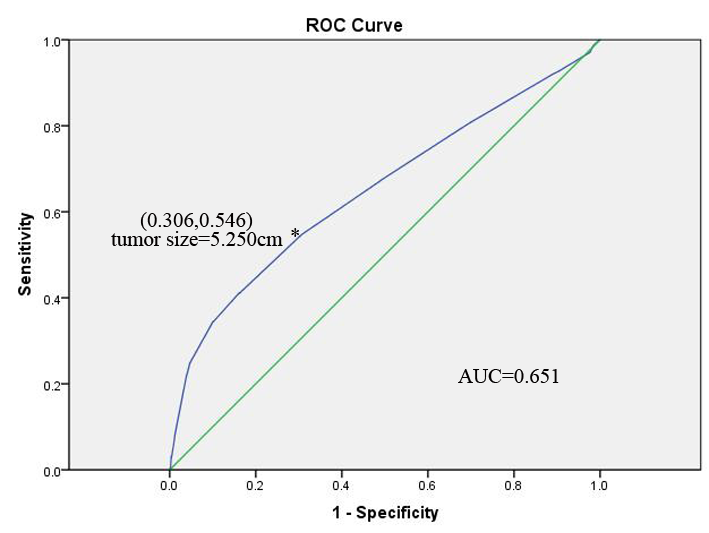

Supplement: Supplementary Figure 4 — Receiver operating characteristic (ROC) curve for tumor size. *The optimal cut-off point of tumor size was 5.250cm. [file Image_4.tif]
